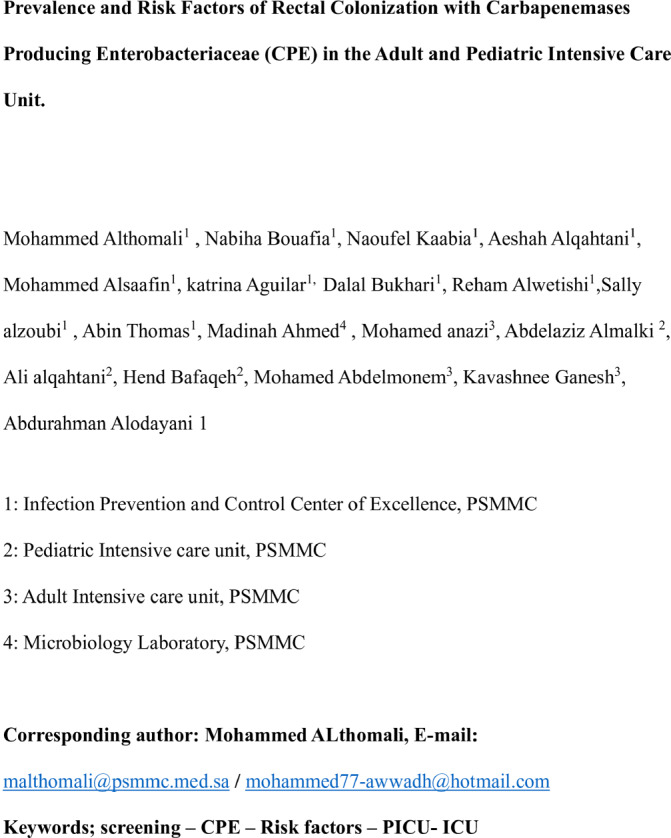# Prevalence and Risk Factors of Rectal Colonization with Carbapenemases Producing Enterobacteriaceae (CPE) in the Adult and Pediatric In

**DOI:** 10.1017/ash.2025.356

**Published:** 2025-09-24

**Authors:** Mohammed Althomali, Nabiha Bouafia, Naoufel Kaabia, Aeshah Alqahtani, Mohammed Alsaafin, katrina Aguilar, Dalal Bukhari, Reham Alwetishi, Sally Alzoubi, Abin Thomas, Mohammed Alanzi, Ali Alqahtani, Hend Bafaqeh, Mohamed Abdelmonem, Kavashnee Ganesh, Najah Alshammary, Abdurahman Alodayani

**Affiliations:** 1 Prince Sultan Military Medical City; 2 PSMMC

## Abstract

**Background:** The prevalence of Carbapenemase-Producing Enterobacterales (CPE) has been increasingly reported worldwide in the past 10 years, representing a serious threat to public health. CPE has become a considerable concern in treatment and infection prevention and control. This study aims to determine the prevalence and risk factors of CPE colonization in the Adult and Pediatric intensive care units (ICUs) in one of the largest military hospitals in Saudi Arabia. **Method:** A cross-sectional study included all new patients admitted to Adult and Pediatric ICUs from December 2022 until December 2023. Rectal swabs were collected upon admission, and CPE was screened using molecular tests (CARBA-R). Demographic, medical-surgical history and clinical data were recorded and analyzed using SPSS 20.0. Risk factors were determined through Multivariate analysis with logistic regression. Odds ratio (OR) confident intervals (CI 95%) were calculated, and a P value less than 0.05 was considered statistically significant. **Result:** A total of 994 patients were included, 70.7% (n=693) of whom were from the Adult ICU. The prevalence rate of CPE colonization was 18.5%, and most cases were recorded in adult ICU (84.4% versus 15.6%; p Keywords; screening – CPE – Risk factors – PICU- ICU

**Figure:**